# Household costs and care seeking patterns associated with COVID-19 in Blantyre, Malawi

**DOI:** 10.1371/journal.pgph.0002003

**Published:** 2023-06-26

**Authors:** Jobiba Chinkhumba, Samuel Mpinganjira, Andrew Kumitawa, Mercy Malopa, Dalitso Longwe, Vincent Samuel Phiri, Tonney S. Nyirenda, Victor Mwapasa

**Affiliations:** 1 Department of Health Systems and Policy, School of Global and Public Health, Kamuzu University of Health Sciences, Blantyre, Malawi; 2 Health Economics and Policy Unit, Kamuzu University of Health Sciences, Blantyre, Malawi; 3 Department of Epidemiology and Biostatistics, School of Global and Public Health, Kamuzu University of Health Sciences, Blantyre, Malawi; 4 Department of Pathology, School of Medicine and Oral Health, Kamuzu University of Health Sciences, Blantyre, Malawi; Jawaharlal Nehru Medical College, INDIA

## Abstract

Economic consequences of COVID-19 illness and healthcare use for households in low income countries are not well known. We estimated costs associated with COVID-19 care-seeking and treatment from a household perspective and assessed determinants of treatment costs. A cross-sectional household survey was conducted between December 2020 and November 2021 in urban and peri-urban areas of Blantyre district. Adults (age ≥18 years) with confirmed COVID-19 were asked to report the symptoms they experienced or prompted them to seek COVID-19 tests as well as healthcare seeking behaviors preceding and following COVID-19 diagnosis. For individuals who sought healthcare, information on out-of-pocket expenditures incurred while seeking and receiving care including on transport, food etc. by both the patients and their guardians was collected. Finally, data on time use seeking, receiving care and during convalesces was recorded. Multivariate Generalized Linear Models were used to evaluate association between household COVID-19 costs and their determinants. Of 171 individuals who took part in the study, the average age was 40.7 years, standard deviation (SD) 15.0, and 50.8% were females. Most participants (85.3%) were symptomatic. Of these, 67.8% sought care at health facilities and the majority (91.7%) were treated as outpatients. The average total household cost associated with COVID-19 seeking, receiving care and convalescence was $62.81 (SD $126.02). Average costs for outpatient and inpatient cases were $52.96 (SD $54.35) and $172.39 (SD $407.08), respectively. Average out-of-pocket household expenditures were $42.62 (SD $123.10), accounting for 62% of total household costs. Being a male COVID-19 patient and engagement in formal employment were significantly associated with high COVID-19 household costs. Households face high economic burden related to COVID-19 sickness and healthcare use. Social policies that support households cope with both the direct and indirect COVID-19 cost are needed to ensure access to healthcare and protect households from COVID-19 related shocks.

## Introduction

Globally, the negative impact of COVID-19 pandemic on economic growth and resource mobilization is substantial [1]. In 2020, the World Bank estimated that the Sub-Saharan Africa region alone was likely to lose between US$37 and US$79 billion in output due to the coronavirus crisis [2]. As countries declared states of emergency and issued stay-at-home orders, hundreds of millions of individuals in low income countries found themselves out of work, both in the formal and informal labor markets [3]. In Malawi, low tax revenues due to the pandemic were estimated to reduce the share of tax revenues from 16.3% of Gross Domestic Product (GDP) in Financial Year (FY) 2019/20 to 15.8% in FY 2020/21, equivalent to a third of government expenditure on health [4]. This underscores that expenditures on health and other social sectors are likely to shrink. Given the pandemic nature of COVID-19, additional investments in the health system are, and will continue to be, required to prevent further spread of the disease, to provide care for the sick and to maintain the provision of other essential services. Therefore, estimates of resources required to provide COVID-19 related services are needed to enable governments and policy makers to better understand and plan for health service delivery [5].

There is little but emerging evidence on the costs of providing care for COVID-19 patients from low income countries [5–7]. Though this information is useful for budgeting amidst shrinking expenditures on health, it is limited in that it mainly comes from the provider or Ministry of Health (MoH) perspective. In other words, it does not consider nor provide insight about households as potential financiers of COVID-19 care. Yet, studies have demonstrated the potential crowding out effects of household health payments on consumptions and on the risk for catastrophic health expenditures [8–10]. In Malawi, households contributions towards total health expenditure accounts for 12% and this contribution has been increasing consistently since 2012/13 [11].

This evidence gap related to the costs of treating COVID-19 patients from household perspective has important ramifications. First, omitting household costs in planning would systematically underestimate, from a societal perspective, the full economic costs of treating COVID-19 which has the potential to lead to sub-optimal resource allocation decisions [12]. Second, given that most adults in low income countries work in the informal sector and have no wage loss insurances [13], not capturing households contributions towards health financing would fail to recognise socio-economic barriers individuals face in accessing COVID-19 treatment. Lack of this critical information would lead to inability to design and implement context specific interventions for comprehensive mitigation of the broad spectrum of COVID-19 consequences at community level in ways that ensure both optimal access to COVID-19 care and financial risk protection for affected households.

Malawi has a GDP per capita income of US$ 381 [14]. Malawi’s health sector financing comprises general revenue, donor funding and household expenditures in the form of direct payments by patients and private healthcare insurers [15]. Latest estimates of Malawi’s general government health expenditure as a share of general government expenditure is 11.4% [16], less than the 15% target set by heads of African Union countries in Abuja [17]. Donor contributions account for 62% of total health funding [14].

Malawi declared a state of national disaster due to the COVID-19 pandemic on 20th March 2020 and registered its first confirmed coronavirus case on 2^nd^ April 2020 [18]. Following World Health Organization guidelines aimed at reducing spread of the virus and resulting socio-economic consequences, the country implemented control measures including disease surveillance systems, dissemination of public health information, ban on public gatherings, closure of schools and national borders and strengthening healthcare facilities for clinical case management [19]. To date, Malawi has experienced four COVID-19 waves resulting in around 88,000 cases and 2, 680 deaths as of 27^th^ September 2022 [20].

As part of a study assessing COVID-19 transmission dynamics within the household, we sought to address the following objectives: a) to estimate costs associated with COVID-19 care-seeking and treatment from a household perspective and b) to assess determinants of COVID-19 household treatment costs.

## Methods

### Study setting

Blantyre is one of Malawi’s four commercial districts. The district has a population of 800, 264 [21] and is in the southern region of Malawi. There are 23 public Health Centers and 93 private for profit clinics of various sizes in the district [22]. Blantyre district is also home to Queen Elizabeth Hospital, Malawi’s largest public tertiary referral facility where the Kamuzu University of Health Sciences (KUHeS) teaching hospital is based and 3 other large private hospitals. These facilities offer COVID-19 admissions and specialized care.

Blantyre district, like the rest of the country, was amid the second COVID-19 wave when this study was conducted. The district has been the epicentre of COVID-19 epidemic in the country reporting 24,313 cases and 684 deaths as of 30^th^ September 2022 [23]. It is likely these reported figures underestimate the true burden of COVID-19 as studies indicate COVID-19 sero-prevalence of 93.9% in the district [24].

### Study design

The Blantyre district health office established a COVID-19 surveillance systems as part of measures to monitor the spread of COVID-19. All individuals with suspected COVID-19 were referred to the surveillance sites for swab collection. COVID-19 tests were done at KUHeS Molecular laboratory within 48 hours of swab collection to confirm presence or absence of COVID-19 among swabbed individuals using SARS-CoV-2 enzyme-linked immunosorbent assay and real-time reverse-transcription polymerase chain reaction (RT-PCR). Centers for Disease Control (CDC) primers were used for the PCR test. Because COVID-19 test results were not available immediately (taking on average 72 hours), tested individuals were phoned or followed by health workers at their homes to be given test results. This cross sectional household survey leveraged this surveillance system. Trained research assistants obtained information on COVID-19 positive cases and then visiting them in their homes for survey recruitment.

### Study population

Eligible for the household survey were individuals with confirmed positive COVID-19 tests (index cases), age ≥18 years who provided voluntary written consent to participate in the survey. Only one individual was enrolled per household. Recruitment and data collection were done within 14 days of a positive COVID-19 diagnosis being made.

### Sample size

For the main COVID-19 transmission dynamics study, it was estimated that the secondary attack rate (incidence) of SARS-CoV-2 infection would be 70% over 28 days of follow-up among contacts of index cases [25]. Using the Cochran’s sample size calculation formula at 95% confidence, proportion of 70% and margin of error of 5%, the sample size of contacts required was estimated as 323. Considering a refusal/tracing failure rate of 20%, this sample size was adjusted to 404. This sample size was further inflated by a design effect of 2 to account for correlations in outcomes among contacts within residential areas, yielding a sample size of 808 contacts. Considering that secondary attack rates would be measured in 3 discrete strata (low density urban, high density urban and peri-urban) and assuming that each index case would have 4 household contacts, the final estimated sample size for index cases was 162, approximately 54 in each of the 3 strata.

### Study data sources

A structured questionnaire programmed digitally with Open Data Kit software and administered using Samsung Galaxy-Tab-2.0 tablets was used for data collection. Information collected included patients’ demographic characteristics such as age, sex, marital and employment status. Patients were further asked to report the symptoms they experienced or prompted them to consult health providers. These symptoms included fever, shortness of breath, cough, sneezing etc. Participants were also asked to report healthcare seeking behaviors before and following the COVID-19 positive diagnosis. This focused on the use of both formal (facility-based care) and informal (i.e. traditional healers and/or self-medications at home) services. For individuals who sought healthcare, information related to out-of-pocket expenditures (OOP) or any costs incurred while receiving care (e.g. on registrations, consultations, drugs, radiological, COVID-19 and other laboratory fees) and on transport, food etc. by both the patients and their guardians was also collected. Finally, data on time spent seeking and receiving healthcare including for convalesces or productive days off work were recorded.

### Costing approach

Following standard guidelines [26], costs were categorised into three groups: direct medical costs, direct non-medical costs and indirect costs. Direct costs are the values of all resources that constitute health services consumed by patients, in this case, these included consultations and medical items such as drugs, COVID-19 and other diagnostic tests. Direct non-medical costs are all out-of-pocket payments towards non-medical items including transport fees to and from health facilities, food bills during care and expenditures on accommodation, if any. Indirect costs, also referred to as productivity loses or opportunity costs, relate to values of time lost-or missed work and business opportunities- while seeking and receiving healthcare including during the recovery period.

### Valuation of productivity loses

The value of time taken to seek, receive COVID-19 care at facilities and productive days lost during recovery and/ or missed work was estimated using the human capital approach [27]. To have lost time or days due to COVID-19 illness, the patient or guardian must have been prevented from engaging in usual activities (productive or leisure) due to the illness or because they were taking care of a COVID-19 person. That is, our estimates of productivity loses (or opportunity costs) were conditional on individuals forgoing either income generating activity and/or nonmarket activities directly or indirectly due to the COVID-19 illness. For each patient with COVID-19, we quantified and added up lost patient and guardians’ time in days. A good number of patients in our sample were either self/ informally employed or un-employed (43.5%). Given the lack of job specific mean wage information among those formally employed, minimum wages were used to value lost productivity for both the formally and informally employed. Use of a minimum wage for those formally employed would underestimate their wages. In contrast, use of minimum wage for the un-employed or informally employed individuals whose real daily earnings were probably lower than the minimum wage would overestimate productivity loss estimates. We estimated productivity losses (opportunity costs) for each patient as the product of the total time lost (in days) and the daily minimum wage pertaining to the year the data were collected. The minimum wages per day in Malawi Kwacha (MK) were MK1,346.16 and MK 1,923.08 for years 2020 and 2021, respectively [28].

We adjusted costs for inflation using annual Consumer Price Index (CPI) increases from 2020 to 2021 to convert 2020 costs to 2021 values. CPIs for 2020 and 2021 were (454.43) and (484.88), respectively [29, 30]. The 2021 average exchange rate for Malawi Kwacha (MK) 799.52 to 1 United Stated Dollar (US$) was used to convert the local currency into 2021 US$ [31].

### Variables definitions and measurements

In line with the study objectives, the main study outcome was COVID-19 household costs. Total COVID-19 household costs were calculated as the sum of direct medical costs, direct non-medical cost and indirect costs incurred by each household with confirmed COVID-19 patient. The total costs of COVID-19 treatment per patient were further stratified by level of care based on whether patients were treated as an outpatient or inpatient.

Other variables of interest included age, sex, marital status, employment status education attainment, presence of co-morbidity such as Diabetes Mellitus and number of days off work. These variables were selected based on the literature, premised on assumption that they would have some bearing on costs as has been reported in previous costing studies [32–34]. **Table 1** provides definitions and measurement details of these variables.

**Table 1:** Variable measurement and coding.

Variable	Definition, measurement and coding
Age	A continuous variable. Measured in years.
Sex	Binary variable. Coded 1 if male and 0 if female.
Education	Primary education attainment, equivalent to 8 years of schooling, coded 0 Secondary education attainment, equivalent to 4 years of schooling plus any years of schooling post-secondary, coded 1
Marital status	Binary variable. Coded 1 if patient married and 0 if patient not married.
Employment status	Categorical variable. Coded 0 if patient not employed, coded 1 if formally or informally employed.
level of care	Binary variable. Code 1 if COVID-19 care was received as an inpatient, 0 if care received as an outpatient
Days ill or off work	Continuous variable. Measured in days.
Co-morbidity	Binary variable. Coded 1 If patient had any morbidity e.g. Diabetes Mellitus, HIV or high blood pressure. Coded 0 otherwise.

### Analysis

We conducted descriptive statistics to explore the distribution of total costs per patient. As expected the costs were heavily skewed to the right [35]. We therefore used boxplots to identify outliers [34] and handled them using winsorisation [35]. Patient costs with values above the 95^th^ percentile were replaced with those of patients in the 95^th^ percentile but patient costs below the 5^th^ percentile were not replaced with the costs of patients in the 5^th^ percentile given that this was “zero” [36, 37]. This way, we kept the entire sample size while reducing influence outliers would have on estimates of central tendencies.

We then calculated the mean, standard deviation (SD), minimum and maximum values for each of the cost categories of interest in the study (direct medical, direct non-medical and indirect costs).

Given the skewed nature of the study outcome of interest (costs), we made standard assumptions that the data would be heteroscedastic and that errors would be independent [38]. We therefore used a multivariate Generalized Linear Model (GLM) to evaluate the association between total household COVID-19 costs and the key cost-driving attribute (level of care) [5, 7] while controlling for other variables such as age, sex, education attainment, employment status and presence of co-morbidities. GLMs do not require that the dependant variable be normally distributed. The empirical GLM took the form:

$$g(u_{i})=\beta_{0}+\beta_{1}Level_{i}+\beta_{2}X_{i},\;y_{i}∼F$$

Where *μ*_*i*_ denotes the dependent variable of interest (household costs) for every unit (adult individual with confirmed COVID-19), *Level*_i,_ is an indicator variable coded 1 if the individual received care as an inpatient, 0 if care was received as an outpatient, X_i_ is a vector of independent variables outlined in **Table 1**. The estimable parameters of interest are thus: β_0_, a common constant for all observations, β_1_, the effect of level of care (and the main target of inference) and β_2,_ representing a vector of coefficients for X. Based on a modified Park’s test, the GLM was specified with a gamma family distribution and log link, where ln(E(y|x))  =  xβ [39, 40]. The forward selection method was used to enter variables. We started with an empty model, then added relevant variables one by one. We assessed accuracy and robustness of the model specification by performing the link test. The hat squared was not significant, *p value* = 0.50, suggesting no problems with the model specification. Because the model has a log link, the exponential of coefficients should be interpreted as the ratio of arithmetic means [38]. The level of care (outpatient or inpatient) was selected as a proxy of disease severity in line with government of Malawi community COVID-19 treatment guidelines which recommends treatment of non-severe COVID-19 cases in outpatient settings while severe cases are admitted for inpatient care [41]. Given number of COVID-19 patients observed, we bootstrapped the results to estimate parameter standard errors and corresponding 95% confidence intervals [42].

### Patients and public involvement

Patients or the public were not involved in the design, or conduct, or reporting, or dissemination plans of our research.

### Ethical approvals

Ethical approval for the study was provided by College of Medicine Research Ethics Committee (COMREC) protocol number P.05/20/3046. Written informed consent was obtained from all individuals before the start of data collection. All methods were carried out in accordance with relevant guidelines and regulations while conforming to the Malawi Ministry of Health recommendations for the prevention of COVID-19 transmission such as wearing of face masks and social distancing.

## Results

A total of 171 individuals with confirmed COVID-19 took part in the study. The participants had a mean age of 40.7 years (SD 15.0). Just over half (50.8%) were females and most of the participants (73.6%) were married. The rest of the study participants’ attributes are shown in **Table 2**.

**Table 2:** Socio-demographic characteristics of COVID-19 cases in urban and peri-urban areas of Blantyre, N = 171.

Attribute	Percentage
Age (mean, sd*)	40.7 (15.0)
Females (%)	50.8
Married (%)	73.6
*Education attainment*	
Primary (%)	5.2
Secondary (%)	32.9
Tertiary (%)	61.7
*Employment status*	
Formal employment	56.4
Informal employment	24.7
Unemployed	18.8
Urban residents (%)	78.9

*SD: Standard deviation

**Fig 1:**
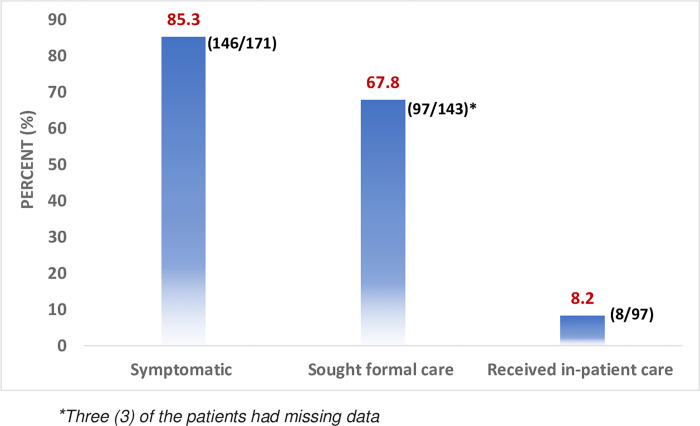
Care seeking patterns for COVID-19 patients in Blantyre, Malawi.

Most of the participants (85.3%) experienced symptoms such as fever, shortness of breath, cough, fatigue and runny nose. Of these, 67.8% sought medical care at a health facility or COVID-19 treatment centres as shown in **Fig 1**. Those admitted spent an average of 3.1 (SD 1.6) days, range: 1–6 days, for in hospital care.

Approximately 15.7% (27/171) of the COVID-19 patients had pre-existing co-morbidities (i.e. Diabetes Mellitus, Hypertension or HIV/AIDS). Of these, 74.0% (20/27) were aware that routine services for individuals with chronic illnesses were being offered at health facilities even though the country was under lock down related to the second wave of COVID-19 infections. During the 6 months before the survey, 65.0% (13/20) of the patients with co-morbidities reported to have sought care at health facilities for their illnesses.

The average total household costs associated with COVID-19 seeking, receiving care and convalescence were $62.81 (SD $126.02). The total outpatient and inpatient costs per household were $52.96 (SD $54.35) and $172.39 (SD $407.08), respectively. Overall, out-of-pocket household expenditures (i.e. direct medical and direct non-medical costs) were $42.62 (SD $123.10). Household costs were consistently lower for outpatients compared with inpatients across the cost categories as shown in **Table 3**.

**Table 3:** COVID-19 household costs (US$) by category and treatment setting.

		Overall					Outpatient					Inpatients			
**Cost category**	**N**	**Mean**	**SD**	**Min**	**Max**	**N**	**Mean**	**SD**	**Min**	**Max**	**N**	**Mean**	**SD**	**Min**	**Max**
Direct medical costs	97	31.80	118.22	0.00	1,125.67	89	21.90	38.43	0.00	193.86	8	141.96	397.48	0.00	1,125.67
Direct -non-medical	97	10.81	26.70	0.00	197.61	89	10.90	27.83	0.00	197.61	8	9.75	6.47	0.00	21.26
Overall OOP	97	42.62	123.10	0.00	1,138.18	89	32.81	52.04	0.00	250.77	8	151.71	398.68	0.00	1,138.18
Opportunity costs	97	20.10	12.19	0.02	72.55	89	20.14	12.13	0.02	72.55	8	20.68	13.72	2.93	40.88
**Total Costs**	**97**	**62.81**	**126.02**	**0.40**	**1,179.07**	**89**	**52.96**	**54.35**	**0.40**	**271.67**	**8**	**172.39**	**407.08**	**7.93**	**1,179.07**

Max = Maximum, Min = Minimum, SD = Standard deviation. All values in the table were estimated after winsorisation.

As shown in **Table 4,** OOP expenditures accounted for most (68%) of household costs associated with COVID-19 care. Wide variation occurred by level of care: OOP expenditures accounted for 62% of costs among outpatients compared with 88% for inpatients.

**Table 4:** Proportion of COVID-19 household costs (US$) by category and treatment setting.

	Overall						Outpatient						Inpatient					
Productivity loses	N	Mean	SD	Median	IQR		N	Mean	SD	Median	IQR		N	Mean	SD	Median	IQR	
Travel time	88	26.3	21.1	20.0	12.5	32.5	81	26.1	21.1	20.0	10.0	30.0	7	27.5	22.8	20.0	15.0	60.0
Waiting / receiving care	89	39.7	50.7	25.0	10.0	60.0	82	39.4	52.7	20.0	8.0	60.0	7	43.5	13.7	45.0	30.0	60.0
**Total time**	**97**	**60.3**	**58.2**	**40.0**	**25.0**	**75.0**	**89**	**60.1**	**59.8**	**40.0**	**25.0**	**75.0**	**8**	**62.2**	**39.0**	**57.5**	**39.0**	**92.5**
Days off work	97	8.2	5.1	8.0	7.0	8.0	89	8.2	5.0	8.0	8.0	8.0	8	8.4	5.8	8.5	3.0	13.0

IQR = Inter quartile range, SD = Standard deviation. All values in the table were estimated after winsorisation.

COVID-19 cases on average spent 60.3 minutes (SD 58.2) seeking, waiting for and receiving care. Time spent did not differ substantially by level of care. Most of the time was spent waiting for and receiving care especially for inpatients (70%) 43.5/62.2 compared to (66%) 39.4/60.1 for outpatients. On average, COVID-19 patients lost 8.2 days (SD 5.1) of their usual work due to COVID-19 illness.

**Table 5** shows the effect of level of care (outpatient or inpatient) on COVI-19 patient costs adjusted for selected control variables. The coefficient for level of care is positive, implying that receipt of inpatent COVID-19 care would be expected to incur higher costs relative to outpatient care, but this is not statistically significant, *p = 0*.*15*. Being male and formally employed were significantly associated with increases in COVID-19 patient costs. Patients’ costs were 80.3% higher for males compared to their female counterparts and were 69.8% higher for cases in formal employment relative to the unemployed. The rest of control variables did not have substantial influence on the COVI-19 patient costs according this model.

**Table 5:** Predicted COVID-19 household costs adjusted for control variables.

	Observed	Bootstrap			
Household costs	Coef.	Std.Err	95% CI		*P value*
Level of care	0.54	0.38	-1.29	1.20	0.15
Age	-0.01	0.01	-0.01	0.02	0.82
Sex	0.59	0.26	0.08	1.16	0.04
Married	0.31	0.31	-0.29	0.93	0.31
Education	-0.37	0.28	-0.92	0.17	0.18
Employed	0.53	0.27	0.01	1.07	0.02
Co-morbidity	0.20	0.37	-0.53	0.94	0.58
Constant	3.66	0.48	2.70	4.61	0.01

Coef = coefficient, Std.err = standard error, 95%CI = 95% Confidence interval. Values estimated using bootstrap

## Discussion

In this study, we estimated household costs associated with COVID-19 treatment for urban and peri-urban households in Blantyre district. The average patient cost for COVID-19 care was $62.81 (SD $126.02). Of this, most $42.62 (SD $123.10) was out- of-pocket expenditure (i.e. direct medical and direct non-medical costs). This amount is more than Malawi’s total health spending per capita, estimated at US$39 per year, which is insufficient to provide essential healthcare as outlined in the country’s health benefit package [4, 43].

Nearly a third (32%) of patient costs were related to indirect or opportunity costs due productive time losses for both the COVID-19 patients and their guardians. These results are consisted with what others have reported that economic consequences for households of illness and healthcare use in low and middle income countries can be a huge burden [39, 44].

Given that for countries in the African region, private health expenditure as a share of total health expenditure is substantial, ranging from 38% for Malawi to 70% in Guinea Bissau [45], these findings underscore that strategies that do not account for individuals’ health financing situations can impoverish households. Because health insurance coverage in low income countries is low [46, 47], the programmatic and policy implications of the findings are that strategies that support households cope with direct and indirect costs of COVID-19 sickness are needed and should underpin all COVID-19 mitigation efforts and social policies.

With respect to determinants of COVID-19 patient costs, the model predicted that male patients and those that are formally employed would be expected to incur higher treatment costs. In general, studies reporting on the relationship between OOP healthcare expenditures and gender provide mixed results. While some studies have reported overall high healthcare expenditures for females compared to men [48], others have documented high expenditures for males [49, 50]. In the local setting, high health expenditures for males probably reflect the fact that males are likely to have severe disease, which would require more service use [51]. Males are also more likely to be formally employed compared to females [52, 53]. This gender differential in healthcare expenditure calls for deliberate efforts to ensure pro-female gender health financing policies in order to promote health for women and reduce inequalities. Gender inequalities in access to health-promoting resources have damaging effects on women’s well-being [54]. The positive association between health expenditures and employment status reflects theoretical postulations that healthcare is a normal good [55] and that those in gainful employment, and by extension greater purchasing power, would demand more healthcare and consequently spend more as well. The lack of association between presence of co-morbidities and costs is rather surprising. Studies have reported higher costs among COVID-19 patients with co-morbidities as they are likely to have complications and require more intensive care [32]. Similarly, one would expect high healthcare costs for those who received inpatient care compared with those treated as outpatients. However, given low number of those with co-morbidities (27 out of 171) and those who received inpatient care (8 out 171) in this study, we cannot rule out lack of power to detect sub-group differences as one reason for these unexpected results. Future studies should therefore have enough power to allow for detailed sub-group analyses.

The majority (85.4%) of COVID-19 patients were symptomatic. This percentage is higher than what others have reported in the region [56, 57]. Despite the high proportion of patients who were symptomatic, only 67.1% sought formal care. This probably stemmed from COVID-19 related travel restrictions, service disruptions and formal communications encouraging those with COVID-19 to self-isolate at home [19, 41]. While lack of knowledge among the COVID-19 cases about where to seek care could also explain this care seeking behavior, it does not appear to have been a major determinant as the majority 74.0% (20/27) of COVID-19 patients with co-morbidities were aware that routine services were available in public health facilities.

The strength of this study is premised on use of laboratory tests to confirm and identify COVID-19 cases. Other costing studies have estimated COVID-19 costs based on reported household expenditures before and after COVID-19 waves in the general populations [58, 59]. In addition, information about costs was collected within 14 days of COVID-19 diagnosis, minimising recall bias challenges [60] and enabling collection of more complete and accurate expenditure estimates.

This study has some limitations. The first relates to the short period of data collection. There is increasing evidence that some of COVID-19 patients experience sequelae which necessitate extra healthcare utilization and costs [61, 62]. Because we did not follow up the patients long term, such costs if any, were omitted. This would bias downwards the patients cost estimates. We propose that future costing studies should follow patients over a longer period to ensure costs related to long term sequelae are captured. Second, enrolling participants after testing positive may have altered health care seeking behavior (Hawthorn effect) [63], affecting estimation of the cost of care. Future studies should explore such changes in behavior/ service use and make appropriate adjustments during cost of care estimations. Finally, this study only included patients in urban and peri-urban areas of Blantyre. Thus, these results cannot be generalised to rural areas. Nevertheless, since cost of living is generally high in urban and peri-urban areas compared to rural settings [64] and the urban poor tend to have detrimental health outcomes [65], focusing attention on urban and peri-urban COVID-19 patients is warranted for informing policies to tackle COVID-19 socio-economic burden among city dwellers.

## Conclusion

Understanding household costs associated with seeking and received COVID-19 care can enable governments and policy makers to better understand the health financing circumstances faced by individuals and inform policy and programmatic decisions. Households face high burden related to economic consequences of COVID-19 sickness and healthcare use. Social policies that support households cope with both the direct and indirect costs associated with COVID-19 are needed to ensure access to healthcare and to protect households from COVID-19 related shocks.
